# Impact of combined plaque structural stress and wall shear stress on coronary plaque progression, regression, and changes in composition

**DOI:** 10.1093/eurheartj/ehz132

**Published:** 2019-03-25

**Authors:** Charis Costopoulos, Lucas H Timmins, Yuan Huang, Olivia Y Hung, David S Molony, Adam J Brown, Emily L Davis, Zhongzhao Teng, Jonathan H Gillard, Habib Samady, Martin R Bennett

**Affiliations:** 1 Division of Cardiovascular Medicine, University of Cambridge, Level 6, ACCI, Hills Road, Addenbrooke’s Hospital, Cambridge, UK; 2 Division of Cardiology, Department of Medicine, Andreas Gruentzig Cardiovascular Center, Emory University School of Medicine, 201 Dowman Drive, Atlanta, GA, USA; 3 Wallace H. Coulter Department of Biomedical Engineering, Georgia Institute of Technology and Emory University School of Medicine, 201 Dowman Drive, Atlanta, GA, USA; 4 Department of Bioengineering, University of Utah, 50 S. Central Campus Drive, Salt Lake City, UT, USA; 5 EPSRC Centre for Mathematical and Statistical Analysis of Multimodal Imaging, University of Cambridge, 20 Clarkson Road, Cambridge, UK; 6 Department of Radiology, University of Cambridge, Hills Road, Addenbrooke's Hospital, Cambridge, UK; 7 Department of Engineering, University of Cambridge, Hills Road, Addenbrooke's Hospital, Cambridge, UK

**Keywords:** Plaque progression, Plaque regression, Wall shear stress, Plaque structural stress

## Abstract

**Aims:**

The focal distribution of atherosclerotic plaques suggests that local biomechanical factors may influence plaque development.

**Methods and results:**

We studied 40 patients at baseline and over 12 months by virtual-histology intravascular ultrasound and bi-plane coronary angiography. We calculated plaque structural stress (PSS), defined as the mean of the maximum principal stress at the peri-luminal region, and wall shear stress (WSS), defined as the parallel frictional force exerted by blood flow on the endothelial surface, in areas undergoing progression or regression. Changes in plaque area, plaque burden (PB), necrotic core (NC), fibrous tissue (FT), fibrofatty tissue, and dense calcium were calculated for each co-registered frame. A total of 4029 co-registered frames were generated. In areas with progression, high PSS was associated with larger increases in NC and small increases in FT vs. low PSS (difference in ΔNC: 0.24 ± 0.06 mm^2^; *P* < 0.0001, difference in ΔFT: −0.15 ± 0.08 mm^2^; *P* = 0.049). In areas with regression, high PSS was associated with increased NC and decreased FT (difference in ΔNC: 0.15 ± 0.04; *P* = 0.0005, difference in ΔFT: −0.31 ± 0.06 mm^2^; *P* < 0.0001). Low WSS was associated with increased PB vs. high WSS in areas with progression (difference in ΔPB: 3.3 ± 0.4%; *P* < 0.001) with a similar pattern observed in areas with regression (difference in ΔPB: 1.2 ± 0.4%; *P* = 0.004). Plaque structural stress and WSS were largely independent of each other (*R*^2^ = 0.002; *P* = 0.001).

**Conclusion:**

Areas with high PSS are associated with compositional changes consistent with increased plaque vulnerability. Areas with low WSS are associated with more plaque growth in areas that progress and less plaque loss in areas that regress. The interplay of PSS and WSS may govern important changes in plaque size and composition.

**See page 1423 for the editorial comment on this article (doi: 10.1093/eurheartj/ehz208)**

**Translational perspective**

- Atherosclerotic plaques are subject to both plaque structural stress (PSS) and wall shear stress (WSS). We show that high PSS is associated with compositional changes suggestive of increased plaque vulnerability, while low WSS is associated with overall plaque growth.
- Incorporation of biomechanical analysis into plaque assessment may help identify patients at higher risk of accelerated plaque growth or deleterious changes in plaque composition, and thus in need of more intensive medical therapy and close follow-up.

## Introduction

Atherosclerotic plaques are distributed non-uniformly along the coronary tree,^1^ suggesting that local haemodynamic factors may determine plaque initiation and development. Plaques also show marked differences in composition across small distances^2^^,^^3^ and are highly dynamic structures. Thus, different areas within a plaque show progression or regression and changes in composition,^4–6^ suggesting that local biomechanical forces may influence future plaque behaviour.^7^

Wall shear stress (WSS) is defined as the parallel frictional force exerted by blood flow on the endothelial surface of the arterial wall.^7^ Wall shear stress can modulate endothelial function,^8^^,^^9^ smooth muscle cell turnover,^10^ and inflammatory adhesion molecule expression,^11^^,^^12^ and thus promote atherogenesis. Indeed, low WSS is associated with plaque growth^13^ and both low and high WSS have been associated with increased plaque vulnerability.^14–17^ Plaque structural stress (PSS) is the stress located inside an atherosclerotic plaque as a consequence of vessel expansion and stretch induced by exposure to arterial pressure and is determined by multiple parameters, including plaque size, composition, and luminal geometry.^18^ High PSS is associated with plaque rupture, presentation with acute coronary syndrome and future adverse cardiovascular events.^3^^,^^19^^,^^20^ However, serial intravascular imaging studies have yet to examine the association between PSS and plaque development. Furthermore, studies to date have not examined the impact of WSS on plaque composition in areas within plaques specifically characterized by either progression or regression, processes that may exist simultaneously in a given vessel or plaque, and therefore, important to be studied separately. Finally, no study has reported how combinations of different values of WSS and PSS are associated with progression/regression or changes in composition within a plaque, although both of these factors are linked with future adverse clinical events.^21^ We, therefore, examined how different combinations of WSS and PSS are associated with plaque development and changes in composition.

## Methods

Detailed Methods are provided in the Supplementary material online.

### Patient recruitment

We recruited 40 patients with typical angina or an abnormal non-invasive test at Emory University Hospital who had non-stenotic but significant coronary lesions in left anterior descending arteries (LAD) [plaque burden (PB) ≥40%, with PB defined as plaque and media cross-sectional area divided by external-elastic-membrane cross-sectional area × 100, but <50% stenosis visually by angiography or <70% stenosis with fractional flow reserve >0.80] (Supplementary material online, *Figure S1*). The study protocol required the inclusion of only LAD arteries. All patients provided informed consent and underwent baseline and follow-up virtual-histology intravascular ultrasound (VH-IVUS) over the course of a year. Sequential angiography and intravascular imaging was protocol-driven, and not a reflection of clinical events or worsening angina. Full details of the clinical studies are available at ClinicalTrials.gov (NCT 00576576 and NCT01230892).

### Virtual-histology intravascular ultrasound image acquisition and analysis

Image acquisition was performed with a phased-array 20-MHz Eagle Eye Gold Catheter and s5 Imaging System (Volcano Corp., USA) using an automated motorized pullback at 0.5 mm/s. Absolute areas of VH-IVUS parameters [fibrofatty tissue (FF), fibrous tissue (FT), necrotic core (NC), and dense calcium (DC)] were measured for each VH-IVUS frame. Changes in areas of external elastic membrane (EEM), plaque components, PB, and plaque area (PA) were calculated as follow-up minus baseline values for each VH-IVUS frame (Supplementary material online, *Methods* and *Figure S1*). Data from 40 vessels generated an average of 98 (56–126) [median (interquartile range)] VH-IVUS frames/vessel. A total of 4029 frames were obtained with each frame undergoing biomechanical analysis as described below. The serial remodelling of each VH-IVUS frame was calculated as ΔEEM area (follow-up EEM minus baseline EEM area) divided by ΔPA (follow-up PA area minus baseline PA).^22^ A ratio of >1 was considered as excessive expansive, 0 to 1.0 as compensatory, and <0 as constrictive remodelling. Progression and regression were defined as increase or decrease in absolute PA compared with baseline. Frames were classified as ‘lipid-rich’ if their appearance was consistent with a fibroatheroma, defined as baseline NC > 10% of plaque cross-sectional area. Segmental analysis was also performed after dividing analysed areas into 2 mm segments, and averaging PSS and WSS across the VH-IVUS frames comprising each segment.

### Biomechanical analysis

#### Plaque structural stress

**Figure 1:**
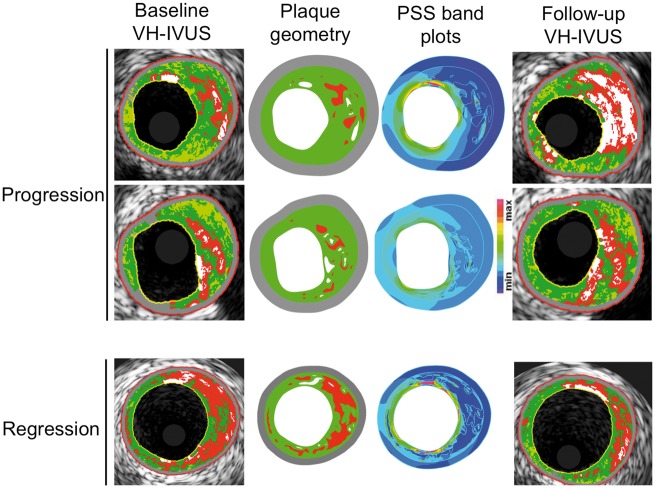
Illustrative examples for stepwise calculation of plaque structural stress from virtual-histology intravascular ultrasound through finite element analysis in areas of progression or regression. Baseline virtual-histology intravascular ultrasound images (left) showing necrotic core (red), dense calcium (white), fibrofatty tissue (light green), and fibrous (green), with associated reconstructed plaque geometry and segmented plaque components used for finite element analysis, and plaque structural stress band plots identifying regions with different stress concentration. Follow-up virtual-histology intravascular ultrasound images (right) show progression (upper and middle panels) or regression (lower panels). VH-IVUS, virtual-histology intravascular ultrasound.

Plaques underwent dynamic 2D finite element analysis (FEA) simulations as previously described^3^ (Supplementary material online, *Methods*). A 65 μm layer of FT was introduced during mesh generation to simulate a fibrous cap, when not present between lumen and NC/DC. Maximum principal stress was used to indicate the critical mechanical conditions within the structure, with PSS defined as the mean of the maximum principal stresses at the peri-luminal region (in essence the circumferential stress calculated on axial frames). Plaque structural stress was subsequently normalized by coronary pressure, creating a ratio for comparison between patients. Examples of baseline VH-IVUS images with their corresponding segmented geometry, PSS band plots and follow-up VH-IVUS images showing progression or regression of plaques are shown in *Figure 1*. Although PSS was calculated in all frames, only those with disease (PB ≥ 40%) were included in the final PSS analysis (1215 frames). Plaque structural stress was categorized into low and high groups with the use of tertiles and on the basis of previous studies such that low PSS = lower 2 tertiles (<5.1), or high PSS = upper tertile (≥5.1).^3^^,^^18^

#### Wall shear stress

**Figure 2:**
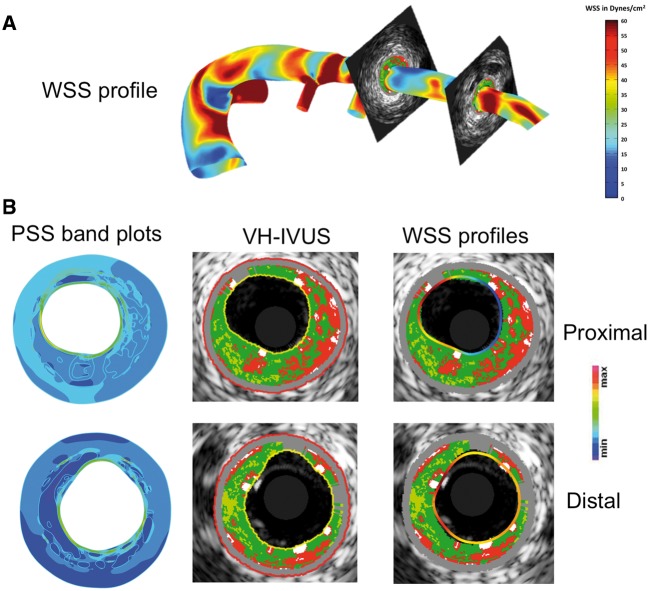
Examples of wall shear stress and plaque structural stress calculations. (*A*) Wall shear stress profile of a left anterior descending artery with (*B*) examples of plaque structural stress band plots (left) from individual virtual-histology intravascular ultrasound frames (middle) and virtual-histology intravascular ultrasound frames with superimposed wall shear stress contours (right) at proximal and distal frames shown in (*A*). PSS, plaque structural stress; VH-IVUS, virtual-histology intravascular ultrasound; WSS, wall shear stress.

Wall shear stress analysis using the ANGUS method has been described previously (Supplementary material online, *Methods*).^13^ As WSS has been implicated in early plaque development, WSS was calculated along the entire length of imaged arteries (4029 frames) irrespective of the presence of atheroma (*Figure 2*). Wall shear stress magnitudes were categorized as low (<10 dynes/cm^2^), intermediate (≥10 and <25 dynes/cm^2^), or high (≥25 dynes/cm^2^) as described previously.^13^

### Statistical analysis

Changes in PB, PA, and plaque composition are presented as median (interquartile range) or mean (standard error of mean). As each plaque had multiple VH-IVUS slices, a linear mixed-effects model was used to account for clustering, with fixed effects for categorical PSS and WSS and a random effect for individual vessels. The change score method was used to allow explicit calculations of changes in area, avoiding under-adjustment of the baseline value and keeping consistency with previous publications. Outliers in the change score were removed using the median absolute deviation method, with the threshold at 3.5 (Supplementary material online, *Methods*). Normal distribution in the linear mixed-effects model was verified on the residuals using quantile–quantile plots. Multivariate analysis was performed on all frames with PB ≥ 40%, irrespective of progression or regression, to further evaluate statistically significant changes in PA, PB, and composition between groups (Supplementary material online, *Methods*). Differences in remodelling (constrictive, compensatory, or excessive expansive) between groups were compared with a χ^2^ test. Intra-observer correlation coefficient assessing absolute agreement was used to assess consistency in PA and composition measurements. All calculations were two-tailed with *P* < 0.05 considered statistically significant. Statistical analyses were performed both in SPSS 21.0.0 (SPSS Inc., IBM Computing, USA) and R 2.10.1 (The R Foundation for Statistical Computing).

## Results

### Baseline patient and virtual-histology intravascular ultrasound characteristics

We analysed LAD arteries in 40 patients by VH-IVUS imaging at baseline and between 6 and 12 months later. Patient characteristics including baseline and follow-up lipid levels are presented in Supplementary material online, *Table S1*. Median baseline PB and PA along the entire vessel length were 32.9% (23.0–45.2%) and 5.12 mm^2^ (2.88–7.72 mm^2^), respectively (Supplementary material online, *Table S2*). Median PB was 51.4% (45.1–59.4%) and PA 8.28 mm^2^ (6.69–10.36 mm^2^) in areas with PB ≥ 40%, confirming that lesions were moderate-sized, and non-obstructive. As expected, diseased areas primarily consisted of FT [2.88 mm^2^ (2.09–4.07 mm^2^)] and NC [1.13 mm^2^ (0.68–1.75 mm^2^)].

**Table 1:** Changes in IVUS characteristics during follow-up

Characteristic	Change over follow-up
IVUS characteristics	*N* = 4029
External elastic membrane (mm^2^)	−0.23 (−0.99 to 0.64)
Plaque area (mm^2^)	0.05 (−0.60 to 0.68)
Plaque burden (%)	0.71 (−3.50 to 5.12)
Necrotic core area (mm^2^)	0.00 (−0.06 to 0.16)
Dense calcium area (mm^2^)	0.00 (−0.01 to 0.07)
Fibrofatty area (mm^2^)	0.00 (−0.09 to 0.03)
Fibrous area (mm^2^)	0.00 (−0.41 to 0.27)
Progression (ΔPA >0) (mm^2^)	*N* = 2099
External elastic membrane (mm^2^)	−0.04 (−0.76–0.77)
Plaque area (mm^2^)	0.65 (0.30 to 1.29)
Plaque burden (%)	4.80 (1.91 to 8.82)
Necrotic core area (mm^2^)	0.02 (0.00 to 0.29)
Dense calcium area (mm^2^)	0.00 (0.00 to 0.09)
Fibrofatty area (mm^2^)	0.00 (−0.01 to 0.08)
Fibrous area (mm^2^)	0.14 (0.00 to 0.66)
Regression (ΔPA <0) (mm^2^)	*N* = 1910
External elastic membrane (mm^2^)	−0.49 (−1.34–0.44)
Plaque area (mm^2^)	−0.62 (−1.21 to −0.28)
Plaque burden (%)	−3.62 (−7.03 to −0.97)
Necrotic core area (mm^2^)	−0.01 (−0.25 to 0.02)
Dense calcium area (mm^2^)	0.00 (−0.05–0.04)
Fibrofatty area (mm^2^)	−0.03 (−0.21–0.00)
Fibrous area (mm^2^)	−0.35 (−0.91–0.00)

Data are presented as median (interquartile range).

No change observed in 20 frames.

**Table 2:** Comparison of changes in plaque characteristics during follow-up between groups

Group	High vs. low PSS			Low vs. high WSS		
	Estimates	CI	*P*-value	Estimates	CI	*P*-value
Entire cohort						
ΔPA (mm^2^)	−0.23 ± 0.08	−0.40 to −0.07	0.006	0.40 ± 0.07	0.27 to 0.54	<0.0001
ΔPB (%)	−0.64 ± 0.40	−1.41 to 0.13	0.102	4.17 ± 0.39	3.40 to 4.95	<0.0001
ΔNC (mm^2^)	0.19 ± 0.04	0.12 to 0.26	<0.0001	−0.01 ± 0.01	−0.04 to 0.02	0.463
ΔDC (mm^2^)	0.03 ± 0.02	−0.01 to 0.07	0.149	−0.01 ± 0.01	−0.02 to 0.00	0.217
ΔFF (mm^2^)	−0.11 ± 0.02	−0.15 to −0.07	<0.0001	0.03 ± 0.01	0.02 to 0.04	<0.0001
ΔFT (mm^2^)	−0.30 ± 0.06	−0.42 to −0.18	<0.0001	0.23 ± 0.04	0.15 to 0.32	<0.0001
Progression						
ΔPA (mm^2^)	0.01 ± 0.07	−0.12 to 0.15	0.832	0.09 ± 0.06	−0.03 to 0.20	0.135
ΔPB (%)	−0.70 ± 0.50	−1.68 to 0.28	0.161	3.28 ± 0.40	2.50 to 4.07	<0.0001
ΔNC (mm^2^)	0.24 ± 0.06	0.13 to 0.36	<0.0001	−0.02 ± 0.02	−0.05 to 0.01	0.179
ΔDC (mm^2^)	−0.02 ± 0.03	−0.08 to 0.05	0.641	−0.001 ± 0.002	−0.01 to 0.00	0.684
ΔFF (mm^2^)	−0.05 ± 0.03	−0.12 to 0.01	0.091	0.02 ± 0.01	0.01 to 0.04	0.004
ΔFT (mm^2^)	−0.15 ± 0.08	−0.30 to −0.00	0.049	0.08 ± 0.04	−0.00 to 0.16	0.051
Regression						
ΔPA (mm^2^)	−0.17 ± 0.07	−0.31 to −0.03	0.018	0.12 ± 0.06	−0.00 to 0.24	0.052
ΔPB (%)	−0.41 ± 0.36	−1.12 to 0.30	0.253	1.16 ± 0.40	0.37 to 1.95	0.004
ΔNC (mm^2^)	0.15 ± 0.04	0.07 to 0.23	0.0005	−0.05 ± 0.02	−0.10 to 0.00	0.053
ΔDC (mm^2^)	0.06 ± 0.02	0.01 to 0.11	0.011	−0.01 ± 0.01	−0.03 to 0.00	0.136
ΔFF (mm^2^)	−0.14 ± 0.02	−0.19 to −0.09	<0.0001	0.02 ± 0.01	−0.01 to 0.04	0.151
ΔFT (mm^2^)	−0.31 ± 0.06	−0.44 to −0.19	<0.0001	0.21 ± 0.06	0.10 to 0.33	0.0004

Results are presented as the difference in mean between groups ± standard error of mean.

Progression and regression are defined as ΔPA > 0 mm^2^ and <0 mm^2^, respectively.

CI, confidence intervals; DC, dense calcium; FF, fibrofatty; FT, fibrous tissue; NC, necrotic core; PA, plaque area; PB, plaque burden; PSS, plaque structural stress; WSS, wall shear stress.

### Changes in plaque size and composition over follow-up

**Figure 3:**
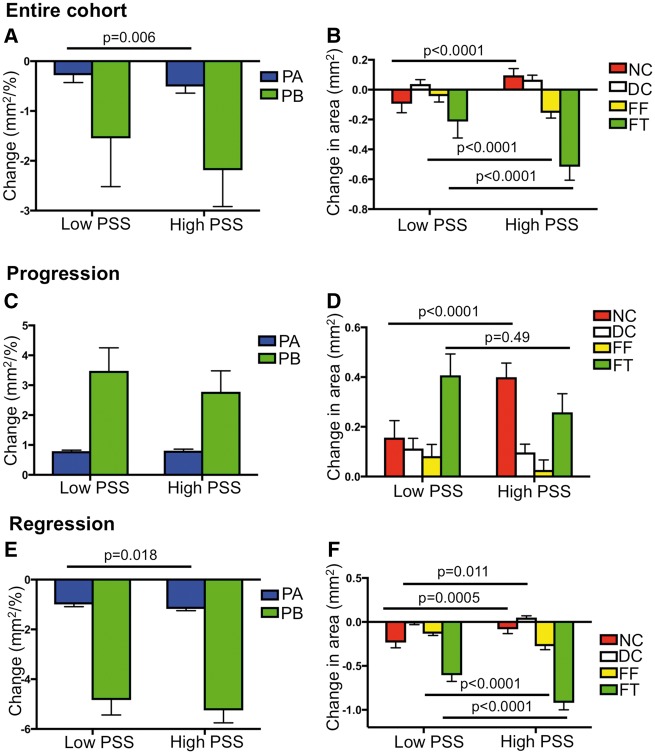
Change in plaque burden, plaque area, and plaque composition according to plaque structural stress. (*A* and *B*) Change in plaque area and plaque burden (*A*) and composition (*B*) in the entire cohort according to plaque structural stress. (*C* and *D*) Change in plaque area and plaque burden (*C*) and composition (*D*) in areas of progression according to plaque structural stress. (*E* and *F*) Change in plaque area and plaque burden (*E*) and composition (*F*) in areas of regression according to plaque structural stress. Data are presented as means ± standard error of mean. Entire cohort (*n* = 1215). *P*-values are for high plaque structural stress vs. low plaque structural stress (only statistically significant values are shown). Figures illustrate the results of linear mixed-effects models (*Table 2*). DC, dense calcium; FF, fibrofatty; FT, fibrous tissue; NC, necrotic core; PA, plaque area; PB, plaque burden; PSS, plaque structural stress.

**Figure 4:**
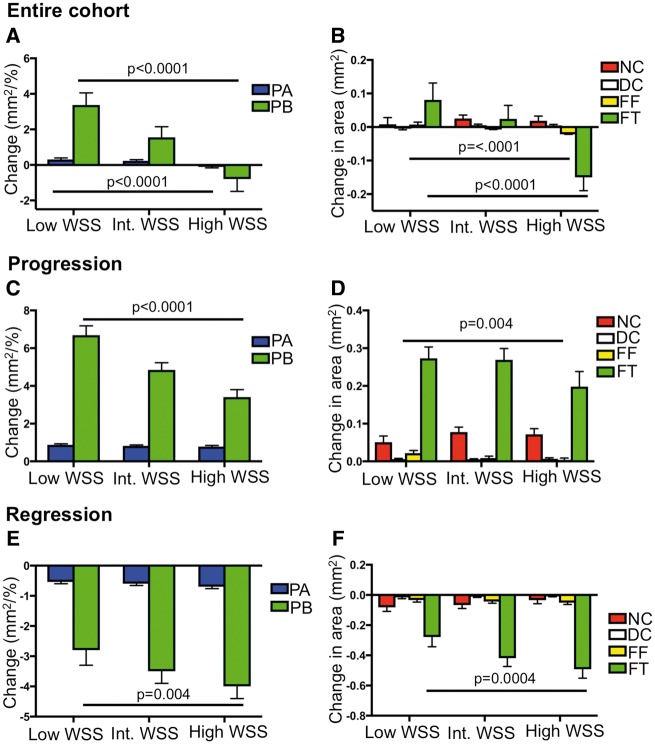
Change in plaque burden, plaque area, and plaque composition according to wall shear stress. (*A* and *B*) Change in plaque area and plaque burden (*A*) and composition (*B*) in the entire cohort according to wall shear stress. (*C* and *D*) Change in plaque area and plaque burden (*C*) and composition (*D*) in areas of progression according to wall shear stress. (*E* and *F*) Change in plaque area and plaque burden (*E*) and composition (*F*) in areas of regression according to wall shear stress. Data are presented as means ± standard error of mean. Entire cohort (*n* = 4029). *P*-values are for low wall shear stress vs. high wall shear stress (only statistically significant values are shown). Figures illustrate the results of linear mixed-effects models (*Table 2*). DC, dense calcium; FF, fibrofatty; FT, fibrous tissue; NC, necrotic core; PA, plaque area; PB, plaque burden; WSS, wall shear stress.

We analysed changes in PB, PA, and plaque composition after baseline and follow-up intravascular ultrasound image co-registration with intra-observer concordance correlation coefficients as follows: PA (0.97), NC (0.98), DC (0.95), FF (0.91), FT (0.95). Although each plaque exhibited multiple different compositional and remodelling changes in different frames at follow-up, overall PA and PB remained largely unchanged (*Table 1*). However, as plaques show increases or decreases in size and composition over time, we also specifically analysed changes in areas of progression (ΔPA > 0 mm^2^) and regression (ΔPA < 0 mm^2^).^6^ Of the 4029 frames analysed, PA increased in 2099 (52.1%) and decreased in 1910 (47.4%), with no change observed in 20 (0.5%) frames. Progression was largely due to increased FT and NC and regression to reduced FT and FF, although there were increases in areas of all components with progression, and decreases in all components except calcification with regression (*Figures**3***and***4*). Excessive expansive remodelling was more common in areas that progressed compared with areas that regressed (39.5 vs. 31.7%; *P* = 0.004), whereas constrictive remodelling was more common in areas that regressed (31.7 vs. 37.1%; *P* = 0.043). There were no adverse clinical events during follow-up.

### Association of plaque structural stress with changes in plaque area, burden, or composition

Finite element analysis was used to estimate PSS in the peri-luminal region in all frames with PB ≥ 40% (*Figure 1*) with PSS subsequently categorized into low and high groups. Plaque structural stress calculations were then mapped to the same VH-IVUS frames (*Figure 2*). ΔPB was similar in high and low PSS areas across the whole cohort, although high PSS was associated with slightly larger decreases in PA (*Figure 3A* and *Table 2*). High PSS was associated with an increase in NC and a larger decrease in FT and FF vs. low PSS, with no differences in ΔDC (*Figure 3B*). Multivariate analysis demonstrated that these associations remained after adjusting for cardiovascular risk factors (Supplementary material online, *Table S3*). Similar results were obtained when ‘lipid-rich’ regions were specifically examined (Supplementary material online, *Results*).

We also assessed whether the relationships observed in the entire cohort were maintained in areas of plaques characterized by either progression or regression. High PSS was not associated with ΔPB or ΔPA vs. low PSS in areas with progression (*Figure 3C*), but high PSS continued to be associated with a larger increase in NC and a smaller increase in FT vs. vs. low PSS (*Figure 3D* and *Table 2*). A similar pattern was observed in areas with regression, with high PSS associated with larger decreases in FT and FF and thus PA as well as smaller decreases in NC (*Figures**3**E *and* F* and *Table 2*). Arterial remodelling was similar between the two groups, irrespective of progression or regression (Supplementary material online, *Figures S2A*–*C*).

To account for any inconsistencies in the co-registration process and compensate for the possibility that adjacent frames may affect the future behaviour of one another, changes in composition, PA and PB across 2 mm segments were also examined (Supplementary material online, *Figure S3*, *Table S4*). Despite the markedly reduced granularity and number of observations, compositional changes similar to frame-based analysis were observed (Supplementary material online, *Figure S3B*, *D*, *F*).

### Association of wall shear stress with changes in plaque area, burden, or composition

As WSS acts on the endothelial surface irrespective of the presence of disease, WSS was calculated across the entire vessel length including in areas with PB < 40%. Low WSS was associated with an overall increase in PB and PA (*Figure 4A*) vs. high WSS, which was largely driven by an increase in FT (*Figure 4B* and *Table 2*). As with PSS, multivariate analysis demonstrated that these associations remained after adjusting for cardiovascular risk factors (Supplementary material online, *Table S3*). Similar results were obtained when ‘lipid-rich’ regions were specifically examined (Supplementary material online, *Results*).

The association between WSS and plaque size and composition was also examined in areas of progression and regression. In areas of progression, low WSS continued to be associated with larger increases in PB compared with high WSS (*Figure 4C*), predominantly due to greater increases in FT (*Figure 4D* and *Table 2*). As WSS acts on the entire artery and low WSS has been suggested to promote early plaque development,^23^ we also examined the association of WSS with plaque changes in areas with PB < 40%. In these areas low WSS was associated with greater increases in PB (4.3 ± 0.5%; *P* < 0.0001), FT (0.16 ± 0.1 mm^2^; *P* = 0.01), and FF (0.09 ± 0.02 mm^2^; *P* < 0.001) but not NC (−0.05 ± 0.04 mm^2^; *P* = 0.12) compared with high WSS. Wall shear stress, like PSS, demonstrated similar associations in areas with regression to those seen in areas with progression. More specifically, low WSS was associated with smaller decreases in PB vs. high WSS (*Figure 4E*) largely due to smaller decreases in FT (*Figure 4F* and *Table 2*). Unlike PSS, significant differences were noted between WSS and arterial remodelling (Supplementary material online, *Figure S4A*–*C*). Areas with low WSS were more likely to undergo constrictive remodelling compared with high WSS in areas of progression with the opposite pattern observed in areas of regression. Conversely, high WSS was more commonly associated with expansive remodelling compared with low WSS in areas of progression with the reverse pattern observed in areas with regression. Analysis across 2 mm segments also demonstrated similar results to the frame-based analysis for changes in PB, PA, and plaque composition associated with different levels of WSS (Supplementary material online, *Figure S5A*–*F*).

### Combination of plaque structural stress and wall shear stress and changes in plaque extent and composition

**Figure 5:**
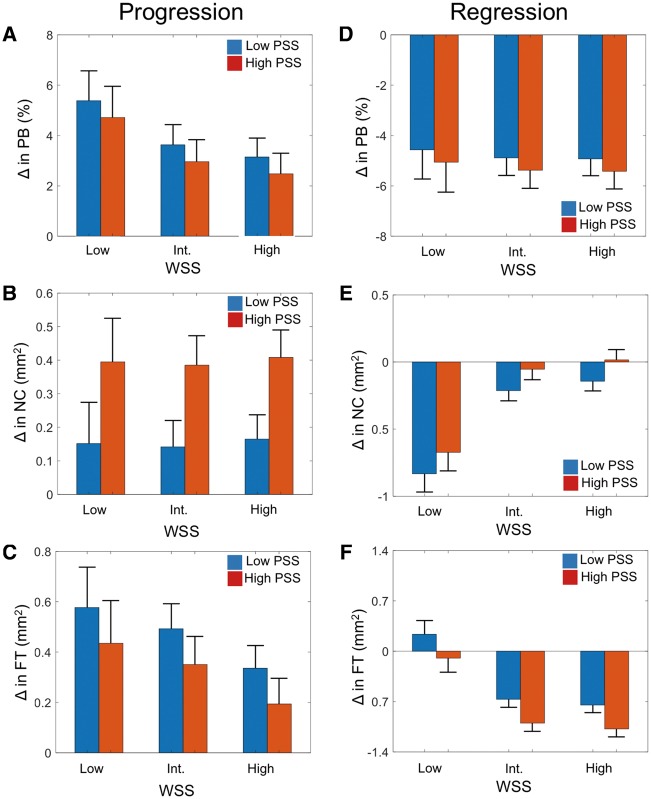
Relationship between plaque structural stress and wall shear stress and plaque change. (*A*–*C*) Change in plaque burden (*A*) necrotic core (*B*) or fibrous tissue (*C*) according to plaque structural stress and wall shear stress group in areas with progression. (*D*–*F*) Change in plaque burden (*D*) necrotic core (*E*) or fibrous tissue (*F*) according to plaque structural stress and wall shear stress group in areas with regression. Data are presented as means ± standard error of mean. FT, fibrous tissue; NC, necrotic core; PB, plaque burden; PSS, plaque structural stress; WSS, wall shear stress (only frames with plaque burden ≥40% included in the analysis).

Plaques are exposed to a number of different forces, including both WSS and PSS. However, how these biomechanical forces interact with each other, and whether their associations with changes in plaque size and composition are independent of each other are not known. Plaque structural stress and WSS were found to be largely independent of each other (Supplementary material online, *Figure S6*) irrespective of PB. We, therefore, examined the association of low, intermediate, and high WSS with low or high PSS on changes in PB, NC, and FT, the latter two features being the major compositional determinants of plaque vulnerability. As both PSS (*Figure 3*) and WSS (*Figure 4*) showed different associations with changes in plaque composition, we examined whether those associations persisted in PAs that showed progression or regression (*Figure**5**A*–*F*).

In areas with progression, lower WSS was associated with larger increases in PB. WSS was not associated with ΔNC, but high PSS continued to be associated with larger increases in NC (added effect of high PSS: 0.24 ± 0.06 mm^2^, *P* < 0.0001) after accounting for WSS. Lower WSS was associated with increased FT, but high PSS showed a non-significant trend towards smaller increases in FT (added effect high PSS: −0.14 ± 0.08 mm^2^, *P* = 0.06) (Figures *5**A*–*C*). In areas with regression, low WSS was associated with smaller reductions in PB, greater reductions in NC, and smaller reductions in FT after controlling for PSS. High PSS was associated with smaller reductions in NC (added effect of high PSS: 0.15 ± 0.04 mm^2^, *P* < 0.0001) but larger decreases in FT (added effect of high PSS: −0.30 ± 0.06 mm^2^, *P* < 0.0001) (*Figure**5**D*–*F*).

**Figure 6:**
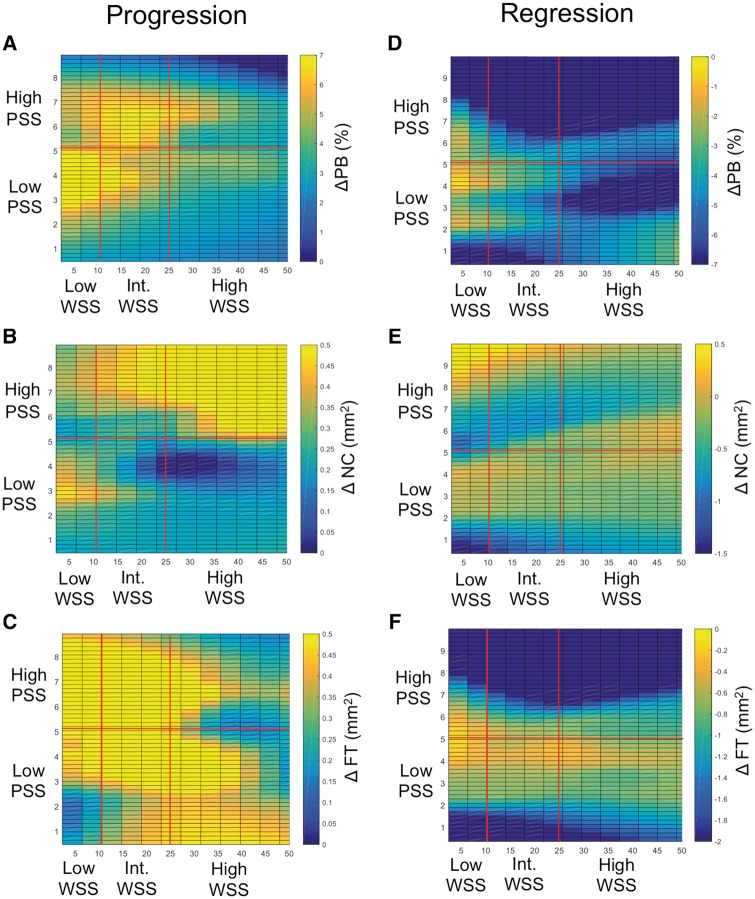
Plaque structural stress and wall shear stress in areas of progression and regression. (*A*–*C*) Heat maps of (*A*) change in plaque burden, (*B*) change in necrotic core, or (*C*) change in fibrous tissue according to wall shear stress and plaque structural stress in areas of progression. (*D*–*F*) Heat maps in areas of (*D*) change in plaque burden, (*E*) change in necrotic core, or (*F*) change in fibrous tissue according to wall shear stress and plaque structural stress in areas of regression. Areas associated with low, intermediate, or high wall shear stress (dynes/cm^2^), and low or high plaque structural stress are marked by red lines. FT, fibrous tissue; NC, necrotic core; PB, plaque burden; PSS, plaque structural stress; WSS, wall shear stress (only frames with plaque burden ≥40% included in the analysis).

As WSS and PSS are continuous variables, we also examined the associations of all levels of WSS and PSS and how they interact on changes in PB, NC, and FT. In areas with progression, the lower the WSS the greater increase in PB, but this was particularly seen in co-localized regions with lower PSS (*Figure 6A*). In contrast, the higher the PSS the greater increase in NC, which was most marked with higher WSS (*Figure 6B*). Increases in FT occurred across many different PSS and WSS regions (*Figure 6C*), although the largest increases occurred with lower WSS and higher PSS, and the smallest increases with higher WSS and PSS (*Figure 6C*). In areas of regression, the lower the WSS the smaller the decrease in PB (*Figure 6D*), irrespective of PSS, and there were no consistent associations between levels of WSS and PSS and changes in NC (*Figure 6E*). In contrast, the higher the PSS the larger the decreases in FT across all WSS (*Figure 6F*).

**Take home figure:**
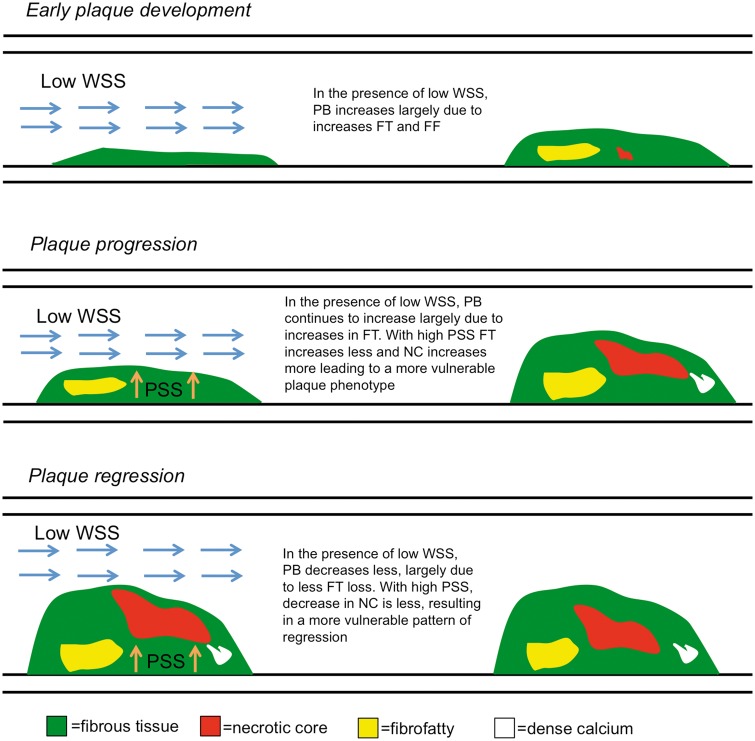
Working hypothesis for the interplay between plaque structural stress, wall shear stress, and future plaque composition. FF, fibrofatty; FT, fibrous tissue; NC, necrotic core; PB, plaque burden; PSS, plaque structural stress; WSS, wall shear stress.

## Discussion

The current study represents the first investigation that (i) evaluates the relationship between baseline PSS and WSS and future plaque composition, (ii) examines the relationship between PSS and WSS, and (iii) evaluates whether the associations observed between PSS and WSS and future plaque growth are independent of the other biomechanical force. We find that baseline PSS is primarily associated with changes in plaque composition irrespective of whether overall change, progression or regression was assessed. Specifically, in areas of progression, high PSS was associated with larger increases in NC and smaller increases in FT, and therefore, evolution to a more vulnerable plaque phenotype. Even in areas of regression, high baseline PSS was associated with larger decreases in FT and smaller decreases in NC. In contrast, WSS is largely associated with plaque growth, defined by changes in PB. Thus, low WSS was associated with a greater increase in PB and/or PA in areas that progress, or smaller decreases in areas that regress, than high WSS. Plaque growth was predominantly due to changes in FT in both very early and more advanced stages of atherosclerosis. In particular, in areas with no or little disease (PB ≤ 40%) low WSS was associated with greater increases in FT than high WSS. Furthermore, we demonstrate that WSS is associated with different patterns of arterial remodelling depending on progression or regression. Low WSS is associated with constrictive remodelling in areas of progression, but expansive remodelling in areas of regression. Thus, our findings demonstrate that the changes in PB observed with low WSS could be due to both changes in PA and the differential pattern of arterial remodelling. Importantly, we also show that there is no significant correlation between PSS and WSS, indicating that their associations with future growth or change in composition are not due to changes in the other stress. Finally, the associations observed between PSS and WSS and plaque change persist irrespective of the other stress, suggesting that different combinations of PSS and WSS may have distinct effects on future plaque behaviour.

The findings of our study are important for a number of reasons. First, they may explain the observation that atherosclerotic plaques are distributed non-uniformly along the coronary tree,^6^ such that both early plaque development and plaque evolution depend on the surrounding milieu, with different PSS and WSS combinations exerting distinctive influences on future plaque composition. Our findings are consistent with mouse and cell culture studies linking low WSS with early plaque development^24–26^ by enhancing expression of inflammatory adhesion molecules and monocyte adhesion, and through induction of pro-atherogenic genes.^27–29^ Second, our findings may explain recent observations that high PSS is associated with future adverse cardiovascular events, as high PSS is associated with progression to more vulnerable plaque phenotypes.^3^^,^^19^^,^^21^ Third, our findings may explain previous studies that have shown that low WSS can be associated with both constrictive and expansive remodelling, as we demonstrate that low WSS is associated with constrictive remodelling in areas of progression, but expansive remodelling in areas of regression.^13^^,^^30^^,^^31^ Finally, our results allow us to speculate on how PSS and WSS may influence future plaque behaviour and composition. In segments with no or limited disease low WSS encourages fibrosis and early plaque development by promoting endothelial cell turnover and inflammatory adhesion molecule expression. Low WSS promotes progression of established plaques, with high PSS promoting a phenotype more prone to rupture. Low WSS also limits plaque regression, with high PSS again promoting a phenotype more prone to rupture (*Take home figure*). Indeed, low WSS has been shown to predict events in the PROSPECT study,^32^ whereas high PSS predicts events in the VIVA study.^3^ The consistency of the associations observed for low WSS and high PSS irrespective of regression, progression, frame or segmental analysis suggests that this combination might be more predictive of future cardiovascular events than either modality alone. These results also suggest that incorporation of biomechanical analysis in plaque assessment may help identify patients that require more aggressive medical therapy to prevent plaque evolution to higher-risk phenotypes.

While our results strongly suggest that baseline PSS and WSS are associated with future plaque behaviour, there are some limitations to our study. First, co-registration of VH-IVUS frames obtained at two different time points can be challenging. However, a second experienced operator independently confirmed accurate co-registration, and additional segmental analysis performed at 2 mm intervals showed similar findings to the frame-based analysis. The latter assumed that PSS and WSS were homogeneous across each 2 mm segment. Second, changes in plaque composition as well as PSS calculations based on VH-IVUS are dependent on its resolution and its ability to accurately identify plaque components. The lateral resolution of VH-IVUS is limited to 200–250μm, such that some changes observed in plaque composition may be either under- or over-estimated. However, VH-IVUS is currently the best intravascular imaging modality for PSS calculations for longitudinal studies as: (i) it offers automatic component identification and segmentation eliminating an important source of human error and (ii) has sufficient penetration to image the whole plaque, which is important for accurate PSS calculations. Third, as several frames were obtained from the same vessel, the effects of clustering may affect the results. However, the effects of clustering were addressed by using a linear mixed-effects model. Fourth, this study examines associations between WSS, PSS and plaque evolution and cannot confirm a cause–effect relationship. However, the consistency of the effects observed for WSS and PSS on plaque size and composition irrespective of regression, progression, frame or segmental analysis strongly suggest that these biomechanical forces directly influence future plaque behaviour. Fifth, this was a small, observational study and as such its results are hypothesis generating with larger studies required to validate these findings.

In conclusion, we demonstrate that areas with high PSS undergo compositional changes suggestive of increased plaque vulnerability. Areas with low WSS demonstrate greater growth in areas of progression and lower plaque loss in areas of regression. These associations with PSS and WSS persist irrespective of the level of the other biomechanical force, suggesting that the interplay between PSS and WSS may have an important role in determining future plaque behaviour.

## Funding

This work was supported by British Heart Foundation [CH/20000003/12800, FS/13/33/30168, and FS/15/26/31441], Heart Research UK [RG2638/14/16], a MRC Confidence in Concepts award, and the NIHR Cambridge Biomedical Research Centre.

**Conflict of interest:** none declared.

## Supplementary Material

Supplementary Data
