# Supplementary material for: Impact of combined plaque structural stress and wall shear stress on coronary plaque progression, regression, and changes in composition
Source: Eur Heart J. 2019 Mar 25;40(18):1411–22. doi: 10.1093/eurheartj/ehz132 (PMC6503452; doi:10.1093/eurheartj/ehz132)
Supplement: Supplementary Data [file ehz132_supp.zip › ehz132-suppl_data/ehz132_Supplemental_Methods_Results_and_Tables_R2.docx]

**Supplement**

# Supplemental Methods

**Patient recruitment**

Details of the clinical studies are available at ClinicalTrials.gov (NCT 00576576 and NCT01230892). To examine whether baseline PSS and WSS are associated with changes in plaque burden (PB), plaque area (PA) and plaque composition in segments characterised by PA progression or regression a cohort of 40 non-consecutive patients presenting with angina symptoms or an abnormal non-invasive test at the Emory University Hospital, Atlanta, Georgia, USA who were found to have non-stenotic coronary lesions on angiography (<50% stenosis by angiography or <70% stenosis with fractional flow reserve >0.80) were recruited^1, 2^. All patients provided written informed consent. Screened patients were excluded only if they did not meet the inclusion criteria or if they declined to participate The study was approved by the Emory University Institutional Review Board. All patients underwent baseline and follow-up radiofrequency backscatter IVUS imaging between 6 and 12 months (Supplemental Figure 1). Coronary flow was assessed at baseline.

Inclusion criteria were:

1. Age > 18 years.
2. Ability to provide informed consent.
3. Left ventricular ejection fraction >30%.
4. The presence of a coronary lesion in the proximal 60 mm of an epicardial vessel deemed significant enough by the operator to warrant further evaluation by VH-IVUS.

Exclusion criteria were:

1. Cardiogenic shock or haemodynamic instability.
2. Lesion requiring percutaneous or surgical revascularization.
3. History of previous coronary artery bypass surgery.
4. Severe valvular heart disease.
5. Presence of visual coronary collaterals.
6. Inability to provide informed consent.
7. Serum creatinine >1.5 mg/dL.
8. Liver disease.
9. Significant haematologic disease.

Cardiac catheterisation was performed via either the radial or femoral route, depending on operator experience and preference. Procedural anticoagulation was maintained using unfractionated heparin (75-100IU/kg), aiming for an activated clotting time of >250 seconds. All patients underwent biplane coronary angiography followed by VH-IVUS. The biplane angles were chosen to visualize the index vessel and at least three horizontal and three vertical markers on the calibration box in each view. Coronary flow was measured with a 0.014-in pressure and Doppler flow velocity monitoring guidewire (ComboWire, Volcano Corp, Rancho Cordova, CA). The ComboWire was first advanced to the tip of the guide catheter, where aortic pressure and guidewire pressures were equalized. This was followed by advancement of the wire into the proximal, non-tortuous portion of the vessel at least 5mm from major angiographic side branches (>2 mm diameter) to measure the inlet velocity. The ComboWire was then advanced into the distal vessel, and the outlet velocity was also recorded. Subsequently, 140μg/kg/min intravenous adenosine was infused for three minutes to induce maximal coronary hyperaemia for the measurement of fractional flow reserve and coronary flow reserve.

IVUS was performed after administration of intra-coronary glyceryl trinitrate with 20MHz Eagle-Eye Gold catheters (Volcano Corporation, Rancho Cordova, California, USA) using a motorized pullback at 0.5mm/s located as distal as possible with a fiduciary side branch used as the starting point. The use of a side-branch as a starting point allowed for the co-registration of baseline and follow-up VH-IVUS images. Radiofrequency data were gathered in diastole at each R-wave peak by patient-specific ECG-gating. All patient and plaque data were entered into a dedicated study database.

**Virtual-histology Intravascular (VH-IVUS) Image Acquisition and Analysis**

Changes in areas of external elastic membrane (EEM), plaque components, PB and plaque area (PA) were calculated as follow-up minus baseline values for each VH-IVUS frame (Supplemental Methods). External elastic membrane (EEM), plaque (plaque and media; EEM minus lumen), and lumen cross-sectional areas were determined for every recorded VH-IVUS frame. PB was calculated as plaque area (PA)/EEM area. Absolute areas of VH-IVUS parameters (fibrofatty tissue (FF), fibrous tissue (FT), necrotic core (NC), and dense calcium (DC)) were measured for each VH-IVUS frame. Baseline and follow-up VH-IVUS images were reviewed side by side for co-registration. The distal end of the target segment was determined by the presence of a reproducible index side branch.

**Biomechanical modeling**

*Plaque Structural Stress Analysis*

Plaque geometry was constructed from VH-IVUS data using an in-house MATLAB code (D3Plaque, Cambridge, UK). Any frames where PB<40% were excluded from biomechanical analysis, as these were considered non-atherosclerotic regions. As *in vivo* data are recorded during diastole, circumferential shrinkage was applied to generate a zero-pressure condition for computational simulation as previously described^3, 4^. Plaque components were assumed to be incompressible, piecewise homogeneous, non-linear isotropic and hyper-elastic as described by the modified Mooney-Rivlin strain energy density function:

$$W=c_{1}\left( \bar{I}_{1}-3 \right)+D_{1}\left[ e^{D_{2}\left( \bar{I}_{1}-3 \right)}-1 \right]+\kappa\left( J-1 \right)$$

where $\bar{I}_{1}=J^{-2/3}I_{1}$ with $I_{1}$ being the first invariant of the unimodular component of the left Cauchy-Green deformation tensor. $J=det\left( \boldsymbol{F} \right)$ and ***F*** is the deformation gradient. $\kappa$ is the Lagrangian multiplier for the incompressibility. $c_{1}$, $D_{1}$ and $D_{2}$ are material parameters derived from previous experimental work^5^ and include; arterial vessel wall, *c*_1_=0.138 kPa, *D*_1_=3.833 kPa, *D*_2_=18.803; fibrous tissue, *c*_1_=0.186 kPa, *D*_1_=5.769 kPa, *D*_2_=18.219 and necrotic core, *c*_1_=0.046 kPa, *D*_1_=4.885 kPa, *D*_2_=5.426. The material properties of dense calcification were derived by fitting a Young’s modulus of 184 MPa derived from experimental work^6^: *c*_1_=1.147×10^5^ kPa, *D*_1_=7.673×10^4^ kPa and *D*_2_=2.838×10^-8^. The motion of each atherosclerotic component is governed by kinetic equations as:

$$\rho v_{i,tt}=\sigma_{ij,j} (i, j=1, 2)$$

where $[v_{i}]$ and $[\sigma_{ij}]$ are the displacement vector and stress tensor, respectively, $\rho$ is the density of each component and $t$ stands for time.

The entire plaque geometric model was meshed using 9-node quadrilaterals (generating approximately 10,000 elements and 40,000 nodes per model). Displacement and strain were assumed to be large. There was no relative movement at the interface of atherosclerotic components and the relative energy tolerance was set to be 0.005. Two adjacent points were fixed to prevent rigid body displacement. PSS was used to characterize the mechanical loading within the plaque structure in the peri-luminal region (0.2mm maximum depth from the luminal contour). Dynamic loading conditions were generated from coronary pressure recordings taken at the time of the procedure. Pressure at the outer boundary was set to zero. All simulations were performed using ADINA 8.6.1 (ADINA R&D, Inc., USA) software.

*Wall shear stress analysis*

The physical 3-dimensional (3D) path of the IVUS catheter during pullback was determined by use of corresponding biplane angiographic projections at least 30° apart acquired before the pullback (IC-PRO, Paieon, Rosh Ha’ayin, Israel). The 3D reconstructed catheter core then served as the stem on which to rebuild the geometry. The 3D position of each ECG-gated IVUS frame was determined from the reconstructed trajectory and speed of catheter pullback. Each frame was aligned perpendicular to the catheter core and adjusted due to rotation. Subsequent to reconstruction of the main artery of interest (left anterior descending coronary artery), arterial branches were added on the basis of information from angiography and IVUS images. The 3D point cloud resulting from the reconstruction was wrapped with a surface (Geomagic Studio, Geomagic Inc., Research Triangle Park, NC) and the surface was meshed in ICEM-CFD (Ansys Inc., Canonsburg, PA). Finally the mesh was imported into the commercial computational fluid dynamics solver Fluent (Ansys Inc., Canonsburg, PA). Inlet and outlet boundary conditions were specified as a series of velocity profiles measured by the Doppler wire. Our assumption for the inlet velocity was a plug profile with velocity equal to 80% of the peak velocity registered in the Doppler ultrasound sample volume (corresponding to peak values in the Doppler spectrum). This profile was imposed at each time step in the pulsatile cycle as determined from the measurements recorded from a stable set of cycles. The inlet section was extended proximally to provide an entrance length of 1 inlet diameter to allow a smooth transition into the computational domain. The fluid (blood) was assumed to be an incompressible Newtonian fluid (viscosity constant with respect to shear rate). It is known that the Newtonian assumption for blood is valid under the pulsatile, moderate Reynolds number flow conditions in coronary arteries. Wall shear stress is dependent on the fluid viscosity and the gradient of the velocity profile perpendicular to the vessel surface. Because viscosity remains constant in Newtonian fluids, WSS is largely dependent on velocity. After the pulsatile flow field in the region of interest was computed, WSS was determined as a function of time in the cardiac cycle and spatial location around the lumen, and the evaluation had previously been shown to be highly reproducible^7, 8^. We next computed the time average of the magnitude of WSS over the pulsatile cycle at each point around the circumference at each axial location (each cross section) and then computed a circumferential average of these values. The result is a spatially and temporally averaged value of WSS magnitude at each axial location.

**Statistical analysis**

Excluded outliers represent changes in composition/plaque area/plaque burden that are much larger than expected in a longitudinal study with follow up over the course of 12 months, irrespective of dose of statin therapy. Such estimated changes are therefore likely to represent artifacts due to inaccuracies in plaque-border detection and tissue classification by the VH-IVUS algorithm, or inaccuracies in the co-registration process in a small number of frames despite the stringent use of anatomical landmarks and review of the co-registration process by an independent second operator. Other co-variates, such as genetic and demographic factors could in theory account for such outliers but this is unlikely to be the case here where follow-up imaging was over the course of a year. However, to verify our conclusions data was also analysed without outlier exclusion (**Supplemental Figures 7 and 8**), with similar results obtained as with the analysis performed following outlier exclusion. Our findings were also verified using other algorithms to robustly fit linear mixed-effects models (R package ‘robustlmm’). The algorithm replaced the residuals and predicted spherical random effects using bounded functions, and employed the design adaptive scale approach for estimation. The results, obtained from iterative reweighting, are highly similar to our findings after outlier exclusion (e.g. ΔNC ~ PSS: 0.19±0.04 vs. 0.19±0.04; ΔFT ~ PSS: -0.28±0.06 vs. -0.28±0.06; ΔPB ~ WSS: 4.17±0.40 vs. 4.14±0.40), thereby demonstrating the robustness of our conclusions.”

Multivariate analysis on all frames with PB≥40, irrespective of progression and regression was performed to further evaluate statistically significant changes in PA, PB and composition between groups. Additional continuous or categorical variables, including age, gender, diabetes mellitus, hypertension, hypercholesterolemia, smoking, family history of CAD, previous myocardial infarction, and previous PCI were considered as potential confounders. For each outcome, covariates that showed p<0.1 in univariate analysis were added to the existing model for PSS/WSS for adjustment.

**Supplemental Results**

**Association of PSS with changes in plaque composition in ‘lipid-rich’ regions**

In ‘lipid-rich’ frames PSS demonstrated similar associations to those observed in the entire cohort. More specifically, high PSS was associated with increased NC (0.11±0.04mm^2^; p=0.01), and greater reduction in FT (-0.34±0.08mm^2^; p<0.0001) and FF (-0.14±0.04mm^2^; p<0.0001).

**Association of WSS with changes in plaque composition in ‘lipid-rich’ regions**

In ‘lipid-rich’ frames, low WSS continued to be associated with an increase in FT (0.17±0.14 vs. -0.16±0.09mm^2^; p<0.0001) but interestingly a larger decrease in NC (-0.24±0.09 vs. -0.02±0.09mm^2^; p<0.001) compared to high WSS.

**Supplemental Tables**

**Supplemental Table 1.** Patient characteristics

| **Characteristic** | **Cohort** |
| --- | --- |
| ***Patient Characteristics*** | ***N=40*** |
| Age, y | 53.4 (11.5) |
| Male, n (%) | 21 (53) |
| Diabetes mellitus, n (%) | 10 (25) |
| Insulin-dependent (%) | 3 (8) |
| Hypertension, n (%) | 29 (73) |
| Hypercholesterolemia, n (%) | 32 (80) |
| Smoker, n (%) | 8 (20) |
| Family history of CAD, n (%) | 18 (45) |
| Previous MI | 5 (13) |
| Previous PCI | 8 (20) |
| **Statin therapy at baseline** | 20 (50) |
| Total cholesterol at baseline (mg/dL) | 168.5 (35.6) |
| LDL cholesterol at baseline (mg/dL) | 97.1 (37.2) |
| HDL cholesterol at baseline (mg/dL) | 44.2 (12.9) |
| Triglyceride levels at baseline (mg/dL) | 117.5 (67.2) |
| **Statin therapy at follow-up** | 40 (100) |
| Total cholesterol at follow-up (mg/dL) | 155.3 (40.4) |
| LDL cholesterol at follow-up (mg/dL) | 85.2 (36.2) |
| HDL cholesterol at follow-up (mg/dL) | 45.7 (13.6) |
| Triglyceride levels at follow-up (mg/dL) | 122.3 (62.0) |
| Data presented as n (%) or mean (SD) | |

**Supplemental Table 2.** VH-IVUS characteristics at baseline

| **Characteristic** | **Cohort** |
| --- | --- |
| ***IVUS Characteristics*** | ***N=4029*** |
| Plaque area, mm^2^ | 5.12 (2.88 – 7.72) |
| Plaque burden, % | 32.9 (23.0 – 45.2) |
| Necrotic core area, mm^2^ | 0.14 (0.00 – 0.88) |
| Dense calcium area, mm^2^ | 0.01 (0.00 – 0.24) |
| Fibrofatty area, mm^2^ | 0.08 (0.00 − 0.34) |
| Fibrous area, mm^2^ | 1.16 (0.00 – 2.64) |
| Data presented as median (IQR) | |

**Supplemental Table 3.** Sensitivity analysis on the entire cohort after adjusting for cardiovascular risk factors.

| **Model** | **Fixed effect** | **p-value** | **Controlled covariates** |
| --- | --- | --- | --- |
| ***ΔPA ~ PSS*** |  |  |  |
| Unadjusted | -0.23±0.08 | 0.006 |  |
| Adjusted | -0.22±0.08 | 0.009 | Age, diabetes and smoking |
| ***ΔNC ~ PSS*** |  |  |  |
| Unadjusted | 0.19±0.04 | <0.001 |  |
| Adjusted | NA | NA | No covariates met p<0.1 |
| ***ΔFF ~ PSS*** |  |  |  |
| Unadjusted | -0.11±0.02 | <0.001 |  |
| Adjusted | -0.11±0.02 | <0.001 | Hypercholesterolaemia |
| ***ΔFT ~ PSS*** |  |  |  |
| Unadjusted | -0.30±0.06 | <0.001 |  |
| Adjusted | -0.30±0.06 | <0.001 | Age and diabetes |
|  |  |  |  |
| ***ΔPA ~ WSS*** |  |  |  |
| Unadjusted | 0.40±0.07 | <0.001 |  |
| Adjusted | NA | NA | No covariates met p<0.1 |
| ***ΔPB ~ WSS*** |  |  |  |
| Unadjusted | 4.17±0.40 | <0.001 |  |
| Adjusted | 4.21±0.39 | <0.001 | Previous myocardial infarction |
| ***ΔFF ~ WSS*** |  |  |  |
| Unadjusted | 0.03±0.01 | <0.001 |  |
| Adjusted | NA | NA | No covariates met p<0.1 |
| ***ΔFT ~ WSS*** |  |  |  |
| Unadjusted | 0.23±0.04 | <0.001 |  |
| Adjusted | NA | NA | No covariates met p<0.1 |
| Results are presented as the difference in mean between groups ± standard error of mean.  PA = plaque area; PB = plaque burden; NC = necrotic core; FF = fibrofatty, FT = fibrous tissue; PSS=plaque structural stress; WSS=wall shear stress.  Fixed effect is measured as high PSS vs. low PSS, or low WSS vs. high WSS. | | | |

| **Characteristic** | **Change over follow-up** |
| --- | --- |
| ***VH-IVUS characteristics*** | ***N=822*** |
| Plaque area, mm^2^ | 0.38 (-1.83 – 2.58) |
| Plaque burden, % | 0.63 (-2.34 – 4.39) |
| Necrotic core area, mm^2^ | 0.05 (-0.43 – 0.80) |
| Dense calcium area, mm^2^ | 0.03 (-0.09 – 0.43) |
| Fibrofatty area, mm^2^ | -0.02 (-0.43 – 0.17) |
| Fibrous area, mm^2^ | -0.04 (-1.59 – 1.01) |
| ***Progression (ΔPA>0), mm^2^*** | ***N=449*** |
| Plaque area, mm^2^ | 2.29 (1.07 – 3.76) |
| Plaque burden, % | 3.81 (1.42 – 7.33) |
| Necrotic core area, mm^2^ | 0.31 (-0.02 – 1.10) |
| Dense calcium area, mm^2^ | 0.10 (-0.02 – 0.49) |
| Fibrofatty area, mm^2^ | 0.03 (-0.18 – 0.35) |
| Fibrous area, mm^2^ | 0.68 (-0.01 – 2.10) |
| ***Regression (ΔPA<0), mm^2^*** | ***N=373*** |
| Plaque area, mm^2^ | -2.01 (-3.69 − -0.96) |
| Plaque burden, % | -2.47 (-4.65 − -0.67) |
| Necrotic core area, mm^2^ | -0.13 (-1.28 – 0.23) |
| Dense calcium area, mm^2^ | 0.00 (-0.19 – 0.30) |
| Fibrofatty area, mm^2^ | -0.17 (-0.91 – 0.03) |
| Fibrous area, mm^2^ | -1.43 (-2.98 – -0.25) |
| Data presented as median (IQR) | |

**Supplemental Table 4.** Changes in IVUS characteristics across 2mm segments

Supplemental References

1. Samady H, Eshtehardi P, McDaniel MC, Suo J, Dhawan SS, Maynard C, Timmins LH, Quyyumi AA, Giddens DP. Coronary artery wall shear stress is associated with progression and transformation of atherosclerotic plaque and arterial remodeling in patients with coronary artery disease. Circulation 2011;**124**(7):779-88.

2. Hung OY, Molony D, Corban MT, Rasoul-Arzrumly E, Maynard C, Eshtehardi P, Dhawan S, Timmins LH, Piccinelli M, Ahn SG, Gogas BD, McDaniel MC, Quyyumi AA, Giddens DP, Samady H. Comprehensive Assessment of Coronary Plaque Progression With Advanced Intravascular Imaging, Physiological Measures, and Wall Shear Stress: A Pilot Double-Blinded Randomized Controlled Clinical Trial of Nebivolol Versus Atenolol in Nonobstructive Coronary Artery Disease. J Am Heart Assoc 2016;**5**(1).

3. Tang D, Teng Z, Canton G, Hatsukami TS, Dong L, Huang X, Yuan C. Local critical stress correlates better than global maximum stress with plaque morphological features linked to atherosclerotic plaque vulnerability: an in vivo multi-patient study. Biomed Eng Online 2009;**8**:15.

4. Huang Y, Teng Z, Sadat U, Hilborne S, Young VE, Graves MJ, Gillard JH. Non-uniform shrinkage for obtaining computational start shape for in-vivo MRI-based plaque vulnerability assessment. J Biomech 2011;**44**(12):2316-9.

5. Teng Z, Zhang Y, Huang Y, Feng J, Yuan J, Lu Q, Sutcliffe MP, Brown AJ, Jing Z, Gillard JH. Material properties of components in human carotid atherosclerotic plaques: a uniaxial extension study. Acta Biomater 2014;**10**(12):5055-63.

6. Ebenstein DM, Coughlin D, Chapman J, Li C, Pruitt LA. Nanomechanical properties of calcification, fibrous tissue, and hematoma from atherosclerotic plaques. J Biomed Mater Res A 2009;**91**(4):1028-37.

7. Jin S, Oshinski J, Giddens DP. Effects of wall motion and compliance on flow patterns in the ascending aorta. J Biomech Eng 2003;**125**(3):347-54.

8. Suo J, Oshinski JN, Giddens DP. Blood flow patterns in the proximal human coronary arteries: relationship to atherosclerotic plaque occurrence. Mol Cell Biomech 2008;**5**(1):9-18.
