# Supplementary figures and images for: Impact of combined plaque structural stress and wall shear stress on coronary plaque progression, regression, and changes in composition
Source: Eur Heart J. 2019 Mar 25;40(18):1411–22. doi: 10.1093/eurheartj/ehz132 (PMC6503452; doi:10.1093/eurheartj/ehz132)

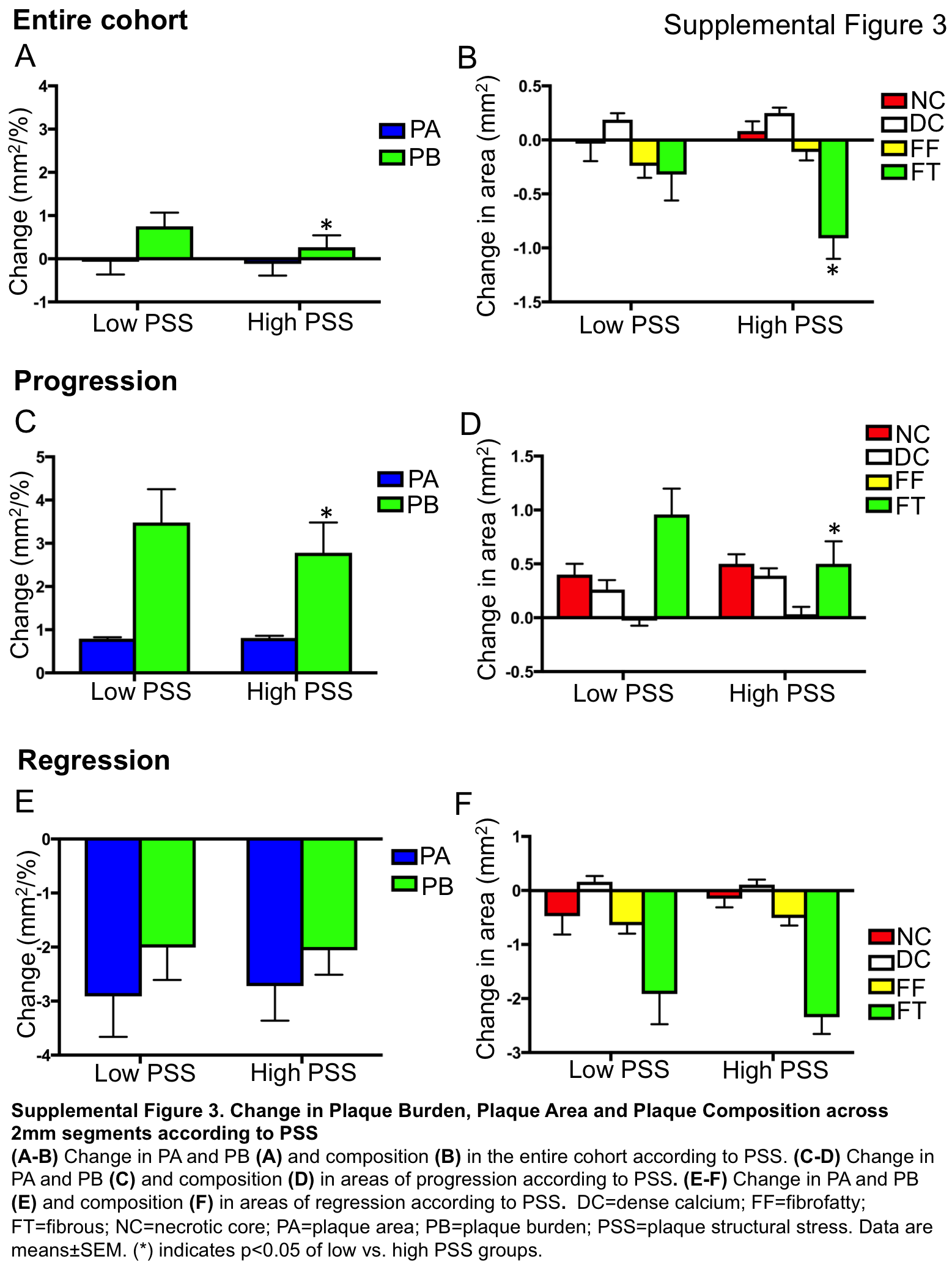

Supplement: Supplementary Data [file ehz132_supp.zip › ehz132-suppl_data/ehz132_Supplemental_Figure_3.tiff]

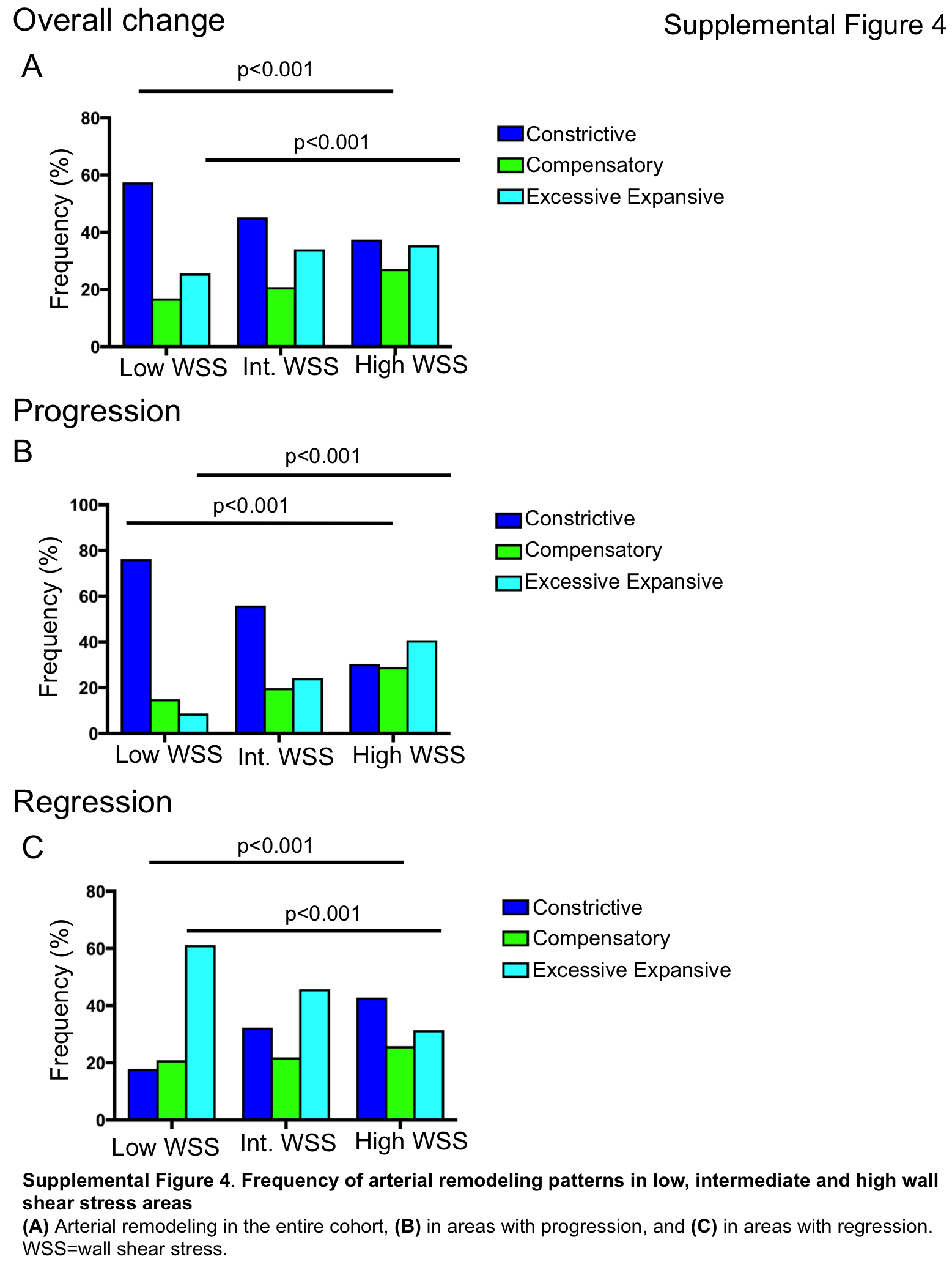

Supplement: Supplementary Data [file ehz132_supp.zip › ehz132-suppl_data/ehz132_Supplemental_Figure_4.tiff]

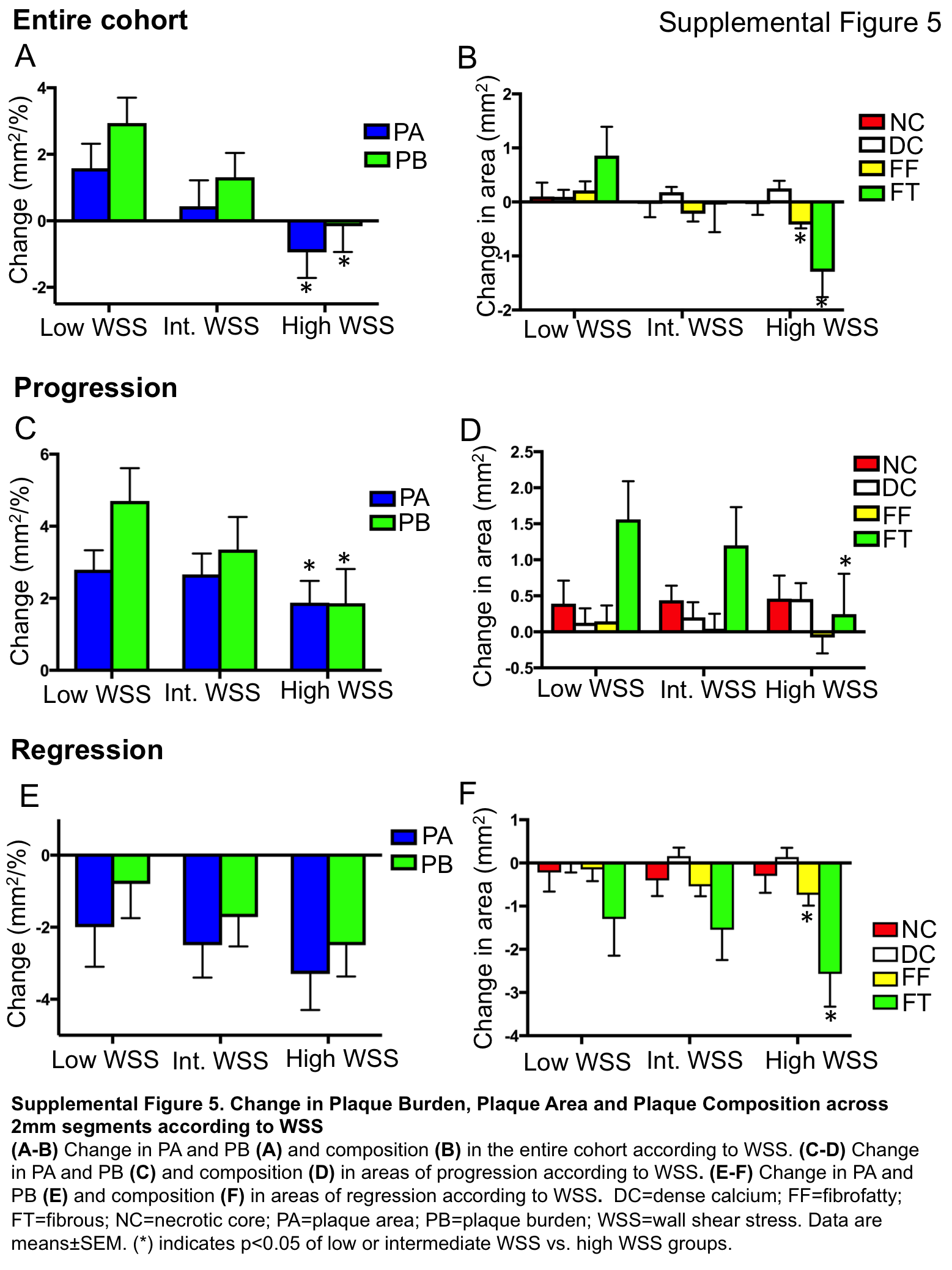

Supplement: Supplementary Data [file ehz132_supp.zip › ehz132-suppl_data/ehz132_Supplemental_Figure_5.tiff]

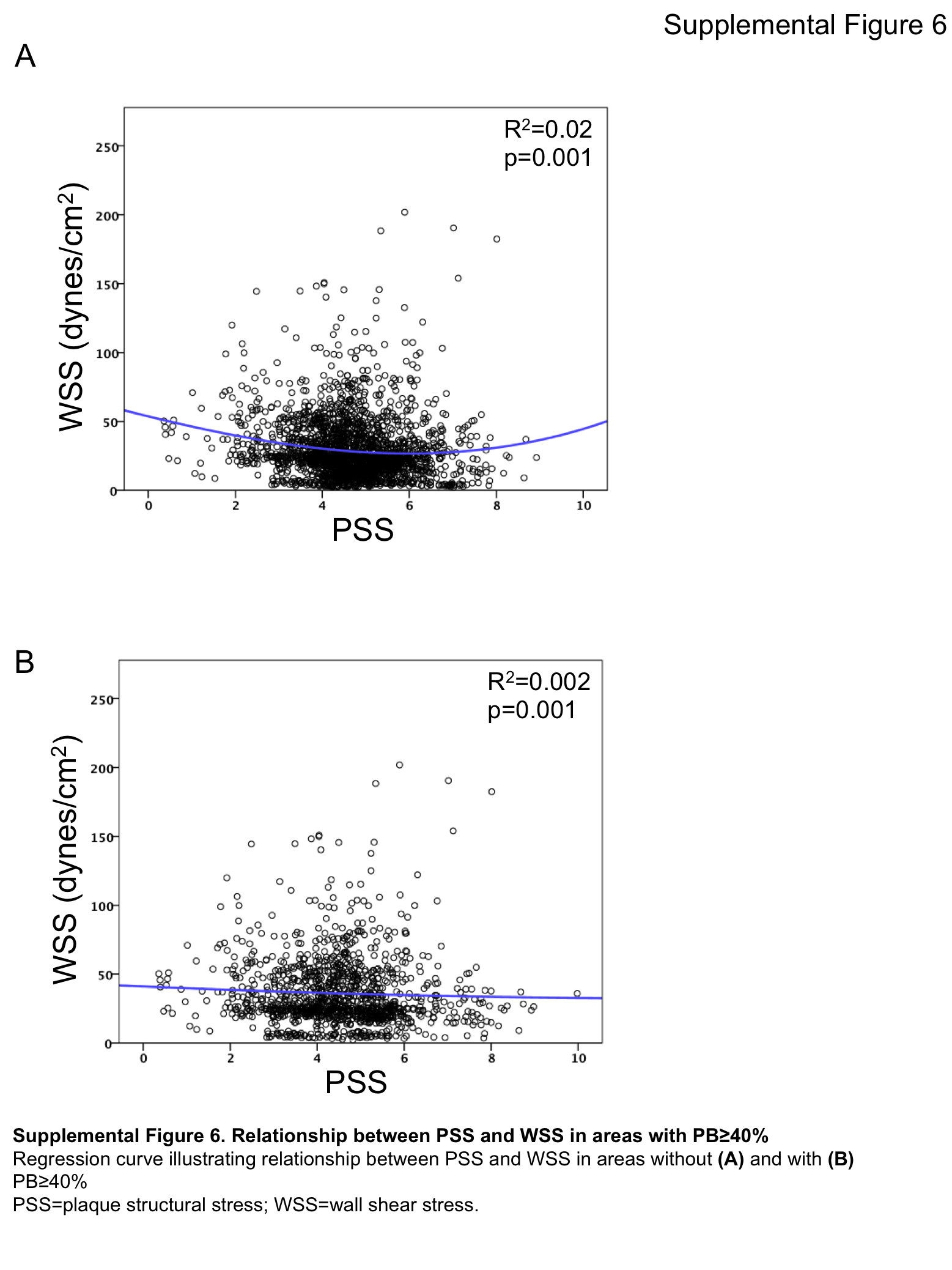

Supplement: Supplementary Data [file ehz132_supp.zip › ehz132-suppl_data/ehz132_Supplemental_Figure_6.tiff]

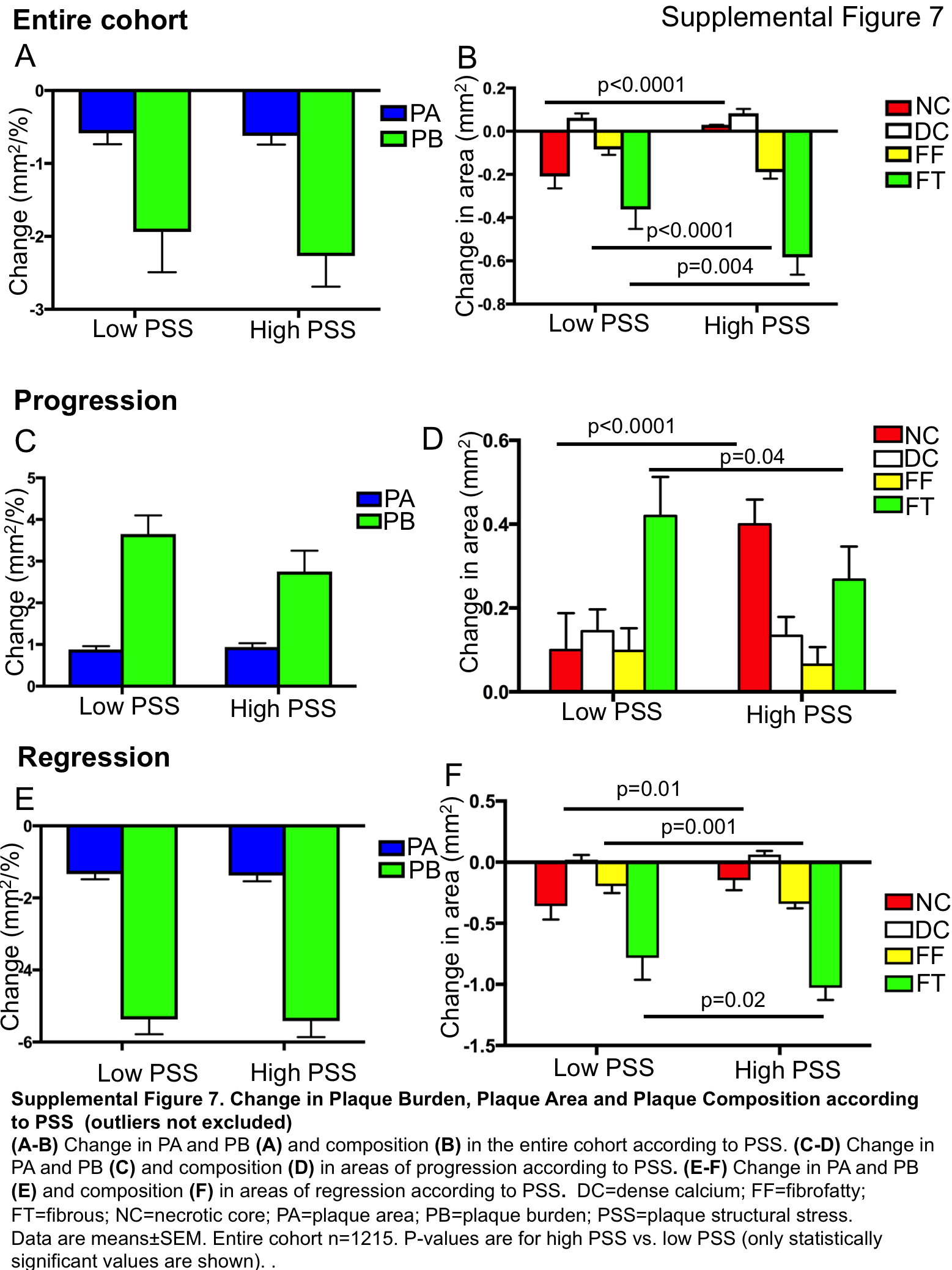

Supplement: Supplementary Data [file ehz132_supp.zip › ehz132-suppl_data/ehz132_Supplemental_Figure_7.tiff]

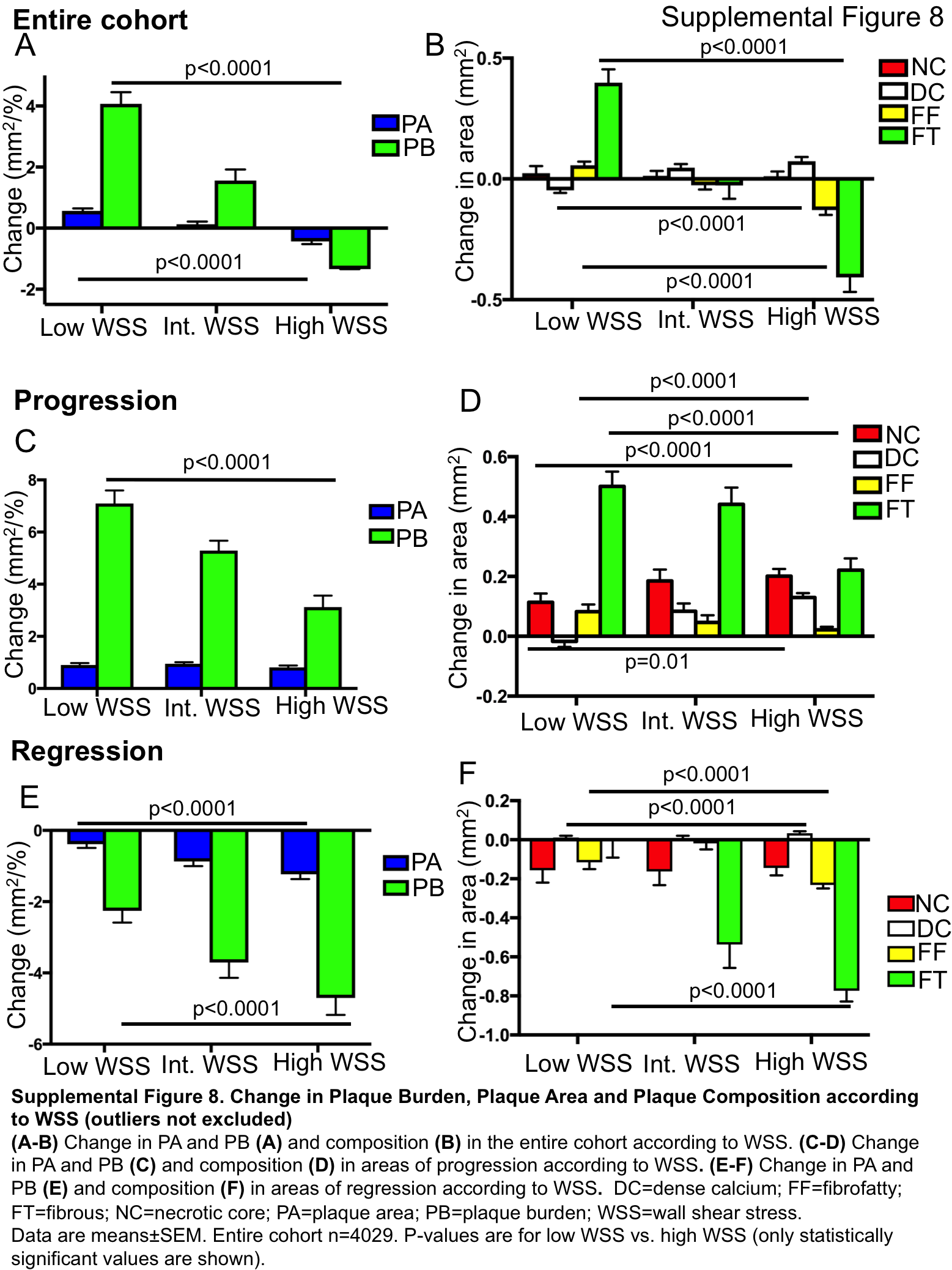

Supplement: Supplementary Data [file ehz132_supp.zip › ehz132-suppl_data/ehz132_Supplemental_Figure_8.tiff]

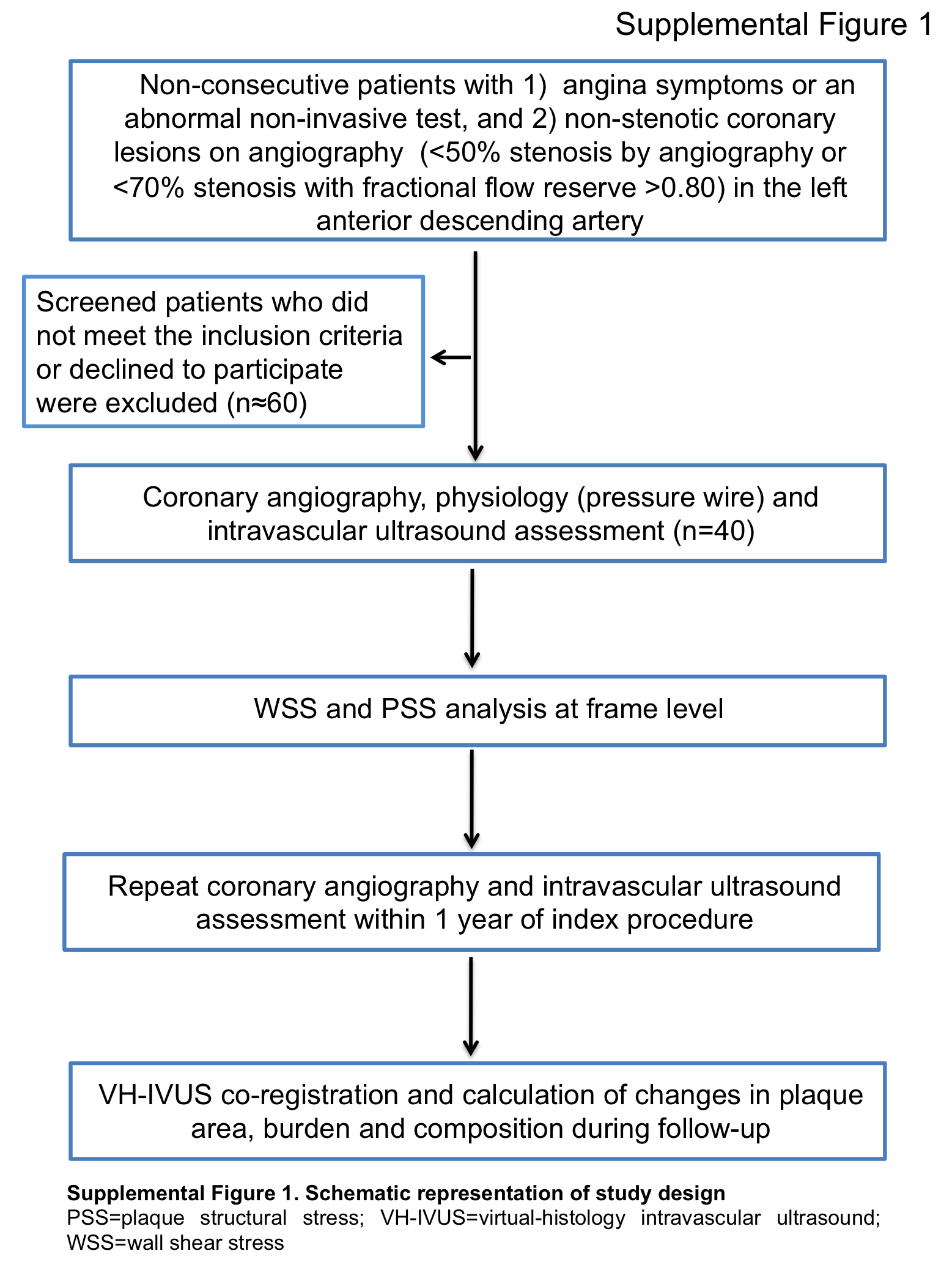

Supplement: Supplementary Data [file ehz132_supp.zip › ehz132-suppl_data/ehz132_Supplemental_Figure_1.tiff]

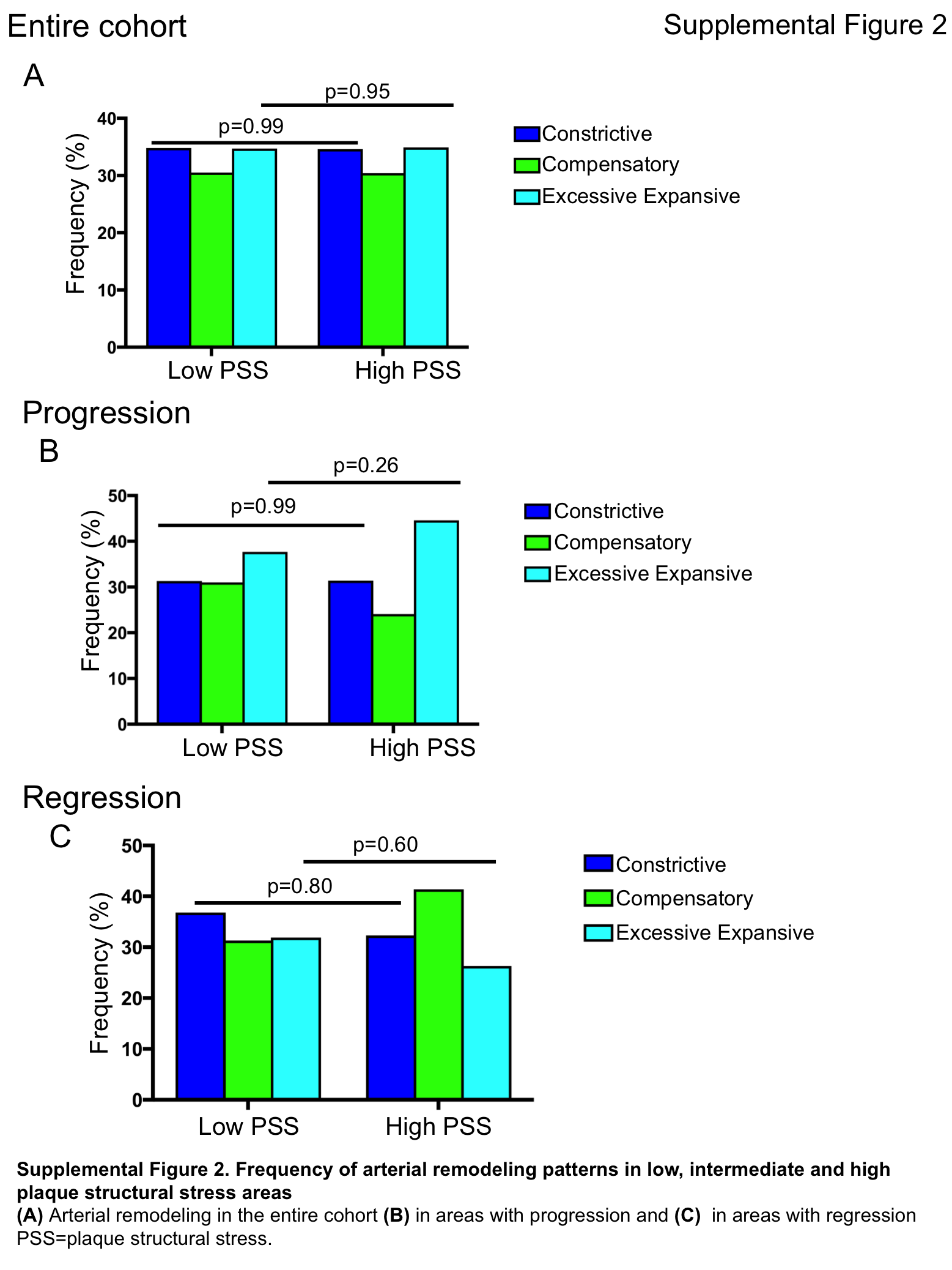

Supplement: Supplementary Data [file ehz132_supp.zip › ehz132-suppl_data/ehz132_Supplemental_Figure_2.tiff]
